# Upgrading Strategies for Managing Nematode Pests on Profitable Crops

**DOI:** 10.3390/plants13111558

**Published:** 2024-06-04

**Authors:** Mahfouz M. M. Abd-Elgawad

**Affiliations:** Plant Pathology Department, Agricultural and Biological Research Institute, National Research Centre, El-Behooth St., Dokki, Giza 12622, Egypt; mahfouzian2000@yahoo.com

**Keywords:** biocontrol, encapsulation, molecular tools, nanoformulations, nematode control

## Abstract

Plant-parasitic nematodes (PPNs) reduce the high profitability of many crops and degrade their quantitative and qualitative yields globally. Traditional nematicides and other nematode control methods are being used against PPNs. However, stakeholders are searching for more sustainable and effective alternatives with limited side effects on the environment and mankind to face increased food demand, unfavorable climate change, and using unhealthy nematicides. This review focuses on upgrading the pre-procedures of PPN control as well as novel measures for their effective and durable management strategies on economically important crops. Sound and effective sampling, extraction, identification, and counting methods of PPNs and their related microorganisms, in addition to perfecting designation of nematode–host susceptibility/resistance, form the bases for these strategies. Therefore, their related frontiers should be expanded to synthesize innovative integrated solutions for these strategies. The latter involve supplanting unsafe nematicides with a new generation of safe and reliable chemical nematicidal and bionematicidal alternatives. For better efficacy, nematicidal materials and techniques should be further developed via computer-aided nematicide design. Bioinformatics devices can reinforce the potential of safe and effective biocontrol agents (BCAs) and their active components. They can delineate the interactions of bionematicides with their targeted PPN species and tackle complex diseases. Also, the functional plan of nematicides based on a blueprint of the intended goals should be further explored. Such goals can currently engage succinate dehydrogenase, acetylcholinesterase, and chitin deacetylase. Nonetheless, other biochemical compounds as novel targets for nematicides should be earnestly sought. Commonly used nematicides should be further tested for synergistic or additive function and be optimized via novel sequential, dual-purpose, and co-application of agricultural inputs, especially in integrated pest management schemes. Future directions and research priorities should address this novelty. Meanwhile, emerging bioactivated nematicides that offer reliability and nematode selectivity should be advanced for their favorable large-scale synthesis. Recent technological means should intervene to prevail over nematicide-related limitations. Nanoencapsulation can challenge production costs, effectiveness, and manufacturing defects of some nematicides. Recent progress in studying molecular plant–nematode interaction mechanisms can be further exploited for novel PPN control given related topics such as interfering RNA techniques, RNA-Seq in BCA development, and targeted genome editing. A few recent materials/techniques for control of PPNs in durable agroecosystems via decision support tools and decision support systems are addressed. The capability and effectiveness of nematicide operation harmony should be optimized via employing proper cooperative mechanisms among all partners.

## 1. Introduction

The recent and quick increase in the world’s population growth rate should be met by securing sufficient food and non-food agricultural production. In this context, nematode pests of many crops represent one of the current pressing issues [1,2,3]. Avowedly, plant-parasitic nematodes (PPNs) can inflict considerable damage to these crops, leading to 13.5% annual losses, equaled USD 358.24 billion, for the top 40 life-sustaining crops alone [2]. Although PPNs are microscopic roundworms, they have a ubiquitous nature, and therefore, remarkable figures are estimated for such losses globally [2,3,4]. Despite various methods that are currently implemented for PPN control, there is consensus that there is no single method that has all the merits of PPN control, i.e., reliable, economical, inclusive, and safe to non-targets [5]. For instance, stakeholders can use resistant genotypes backed by agricultural practices for PPN control. Nonetheless, resistant genotypes and related techniques are often unavailable or inadequate for numerous plant species/cultivars. In addition, the increase in global temperature due to climate change is expected to affect nematode populations either by accelerating their life cycle as the soil temperature increases or by changing host plant physiology, which facilitates the infection process [6]. According to the National Oceanic and Atmospheric Administration (NOAA) global analysis for the year 2017, the 2017 average global temperature across land and ocean surface areas was 0.84 °C above the 20th century average of 13.9 °C (https://www.ncdc.noaa.gov/sotc/global/201713, accessed on 1 June 2024). Therefore, the need for continuous optimization of strategies for managing nematode pathogens of economically important crops is apparent [6]. Such strategies should be based on not only perfections of the pre-procedures of PPN control, such as sampling and extraction techniques, but also exploiting the full spectrum of plant–nematode interactions. Perfecting such pre-procedures is rational and should be followed by applying adequate ratings to define host susceptibility/resistance and consequent/expected crop losses before performing sustainable and effective strategies to control PPNs and fulfill food security. In essence, topics related to such pre-procedures will be briefly addressed herein before discussing current and emerging strategies for wise management of nematode pests on key crops. Adjusted strategies for managing these pests in light of current issues such as climate change should be considered [7]. They necessitate synthesizing superior management strategies via novel approaches, e.g., new biochemical targets of safe and novel nematicides. This review addresses such strategies that will not only delay the pest’s resistance against the novel nematicide but also assist in identifying more selective nematicidal compounds. It extends and updates employing such strategies in order to circumvent the excessive and unwise use of some effective chemical nematicides. Thus, further approaches exemplified herein may include low/no side effects to function synergistically or additively via sequential, dual-purpose, and co-application with other production inputs in integrated pest management plans [8]. This review presents the ongoing progress in the related research and techniques, such as the nanoencapsulation of nematicides as well as bioinformatics and searching RNA-Seq to develop efficient and benign management of PPN pests. Ultimately, a PPN management strategy for modern agronomic and horticultural systems is knowledge intensive. It should combine accurate PPN density and distribution patterns, host suitability designation, damage thresholds, and biocontrol agent (BCA) efficacy as well as sensitivity to the existing biotic/abiotic factors and reliability of any expected management measure(s). Therefore, basic pre-procedures for superior PPN control are initially highlighted herein.

## 2. Sound Sampling and Extraction Methods as Prerequisites for the Strategies

Such methods should be applied with an end in view to fulfill efficient integrated pest management (IPM) within the best available cost-effective schemes. Because sampling and extraction relate to various aspects of PPN research and control, merits and demerits of their methods are linked to all relevant scopes. Truly, PPN sampling and extraction are imperative to isolate, identify, and assess their population levels in soil/plant tissues, but timing, intensity, pattern, tools, and the related materials/microorganisms sampled/extracted all rely on the required goal(s). Therefore, carefully designed schemes are critical to avoid unwanted issues while achieving the target goals with the allocated funds [9].

### 2.1. Avoiding Common Defects in Classical Sampling and Extraction Methods

Initially, researchers involved in PPN sampling should be aware of the reliability (accuracy) and precision levels of their samples in light of the desired objective, location, commodity value, crop susceptibility, PPN species, and devoted funds. Hence, natural PPN spatial/temporal patterns and their impact on nematode frequency distributions are needed in most sampling methods [9,10]. Awareness of factors that can affect such frequency distributions should be considered, e.g., dimension of the sampler tool and type of samples (single vs. composite). Occasionally, confidence intervals of PPN population means are estimated to be well above/below the action threshold levels, which guarantees obtaining fewer samples with reasonable accuracy/precision levels. In contrast, if such measured confidence profiles of PPN population densities are not well above/below their action thresholds, sampling accuracy and precision reflect the need to consider more samples [11]. Sampling reliability/precision levels may be boosted and tailored with the current progress and advancements in processing PPN sampling rate [10] as well as PPN counting [12] to a several folds increase. Likewise, the extraction method chosen should provide the most authentic and relevant soil fauna/flora data [11,13]. Abd-Elgawad [11] presented tables to demonstrate considerably different funds needed to sample two nematode taxa with low and high action thresholds, root-knot nematodes (RKNs) on berseem clover and citrus nematodes on citrus trees, respectively, at various accuracy and precision levels. Surely, precision agriculture should collect, process, and examine these temporal/spatial distribution patterns with related PPN data; e.g., BCAs as well as economic factors such as assessed variability for enhanced resource use efficiency and profitability, in order to back sound decisions for PPN management strategies.

Heavily PPN-infected plants frequently have too small of root systems to host many nematodes, but samples from close and less-infected roots may host more PPNs in their relatively big root systems. Thus, the sampling unit should be carefully chosen. In this context, soil samples taken from the rhizosphere are frequently used to assess PPN number per either weight in g or volume in cm^3^. However, to circumvent the balance between the invaded root densities and PPN numbers, it is best to count PPN number per g of fibrous roots in the same volume of soil. Using such a sample volume and weight simultaneously can avoid flaws in estimating PPN population levels related to plant damage. Such an estimate is a clear aim for sound PPN management strategies. In contrast, using either weight or volume units separately may lead to false correlations between PPN population densities and crop yields/growth parameters.

Sampling should serve its objective: advisory service, survey, research, or correlating PPN population densities to certain ecological/biological factor(s) and/or agricultural practices. As a case in point, both plant roots and soil should be sampled for PPNs at planting time or during pre-planting of seasonal crops for advisory services. Also, samples that relate the PPN population level to particular biological/edaphic factor(s) or agricultural exercises can reliably participate in IPM. Adequate periodical sampling, for example, can trace the efficacy of an introduced or indigenous biological control agent for its further development and activity against the existing PPN population. Moreover, it can also monitor harmful organisms in a polyspecific soil community to block their unfavorable effects against beneficial invertebrates [9,14] in IPM. Sampling at different stages of the plant’s growth can provide evidence of whether the dominance/spread of natural/applied BCA gradually/rapidly drops/increases in each stage. These techniques can properly address IPM for PPNs with various BCAs such as fungi, bacteria, and entomopathogenic nematodes (EPNs). Admittedly, a complementary approach for extracting soil nematodes should utilize some version of Baermann funnels/trays, decanting and sieving, centrifugal flotation, and/or elutriation (semi-automated). For extracting PPN from soil or plant material, a mistifier method to spray an intermittent mist over the sample is a good Baermann adaptation. Also, PPN extraction efficiency can be boosted via exposing macerated roots infected by nematodes, especially RKNs, to instant 0.5% NaOCl for 10 s [15] or by enzymatic predigestion of the infected plant material before mechanical maceration [16]. Yet, both of these efficient methods should be followed by cleaning up the PPN suspension. Therefore, a form of Baermann or centrifugal flotation techniques is applied for mobile or immobile PPNs, respectively. Thus, efficient methods such as enzymatic predigestion and using mistifiers are proper. They form intermediate points in the continuum of advancing PPN sampling, extraction, counting, and finally developing strategies for their management.

### 2.2. Expanding High Throughputs of Nematode Sampling, Extraction, Identification, and Counting

Generally, the more samples collected, sampling times, or bioassays done, the better information obtained but the greater the cost. The longstanding recognition that mechanized devices for PPN sampling, extraction, identification, and counting can offer inexpensive and excellent tools for novel and effective strategies of PPN management remains mostly unexploited in nematode processes [9,17,18,19,20]. Mechanization can enhance the sampling accuracy/precision [10], but it requires a well-trained operator on the relevant equipment (e.g., drive the tractor in a perfect manner, faultless review of the map for the sampled area, and careful handling of the samples/bag holder). Also, mechanical maceration after enzymatic predigestion of the sampled tissues can greatly boost extraction efficiency, but it is rarely used. On the other hand, quantitative real-time PCR (qPCR) approaches are being progressively utilized to supply accurate techniques to identify and quantify nematodes and related soil BCAs as well as disease microorganisms [8,21]. Thus, progress in mechanizing DNA-based analyses to reliably identify and quantify multiple pest and disease organisms should be expanded, as they are gradually becoming easier and more cost-effective approaches [22,23,24]. Using qPCR for identification and quantification of soil organisms, biocontrol, and nematode competitors could be substantially judged via stepwise correlations of variables at various lags of pest stresses [23]. Hence, qPCR can contribute in clarifying soil food webs at a landscape scale for upgrading management strategies of soil pests. Furthermore, nematode species detected by qPCR can also be detected by high-throughput sequencing (HTS) with a high correlation between qPCR and HTS for species with relative abundances [24]. Interestingly, HTS represents a cost-effective and reliable technique for defining soil food webs as well. Moreover, HTS provides a fine-scale taxonomic resolution to differentiate among closely related species of BCAs [25]. This merit can boost biocontrol, especially in IPM schemes, as the matched species will be aptly applied due to exhibiting unlike phenotypes for their key attributes, e.g., host specificity and habitat adaptation. It can also resolve a challenging matter for conservation biocontrol strategies against PPNs because practices aimed at maintaining/enhancing PPN suppression may not be reliable due to exclusive dependence on indigenous antagonists [26]. Factually, molecular identification and quantification of existing/indigenous antagonists can lead to decisions on where it is effective to utilize conservation biocontrol. Similarly, the success of nematologists in extending the frontiers of PPN management-related knowledge should be properly exploited. For example, development of automated image processing for high-throughput nematode counting could accurately and rapidly function for high population levels of BCAs, e.g., entomopathogenic nematodes and other microorganisms [12]. Also, a new technique to extract females of *Tylenchulus semipenetrans* suitable for molecular studies from citrus roots may boost the chances of designating rootstocks with more desired traits [27]. Using molecular techniques and automated workflows in the above-mentioned nematode processes with precision and pace as good as or better than classical methods has been established and should be expanded for better achievements. Moreover, further cost decreases can be attained by technical optimizations in recent methodologies, e.g., designing lower-cost probes and developing multiplex systems to quantify the polyspecific PPN community as well as other microorganisms [9,19,23,24,25]. Such novel technologies have also been used to detect and infer PPN spatial patterns to be harnessed in novel nematode management programs [28]. Incorporating these technologies with indices of nematode spatial/temporal distributions can aptly predict the need for resistant planting material [9] or lead to the precise uses of other control measures [29].

## 3. Proper Gene Pools and Scales Used to Determine Host Susceptibility/Resistance

Host-plant resistance (HPR) to PPNs will continue to be a key tool in optimizing PPN management strategies as long as we try to reconcile the value of boosting crop production with that of healthy lives and environmental conservation. Plant breeding for nematode resistance has several merits. It has been one of the most economical, reliable, and safe methods for increasing food production [8]. The success of breeding programs for PPN resistance, however, depends on three main elements: hereditable genetic variations, reproduction of the selected genotypes, and adequate screening techniques [30]. Utilizing crop sequences, including genetically resistant plant species/cultivars, should help with PPN control were it not for the dearth of PPN-resistant genotypes required in the crop rotation. Hence, novel PPN management strategies via selective breeding should further widen the gene pools of different plant genotypes, as success is likely only if the desired alleles within reproducible genotypes are found in the gene pools. These strategies are further expected to carefully utilize resistant/immune plant genotypes as an integral component of sustainable pest management plans. This integration is especially timely with the occurrence of more virulent PPN isolates, which threaten the usefulness of this resistance. For instance, the appearance of current resistance-breaking pathotypes/isolates of key RKN species has been confirmed to reproduce and damage resistant genotypes of economically important crops such as tomato [31], pepper [32], cowpea [33], and potato [34]. Tackling such problems in new PPN control tactics/strategies is ongoing for further application on large scales. For instance, as wild relatives of a plant species are assumed to grow in more diverse settings than domesticated cultivars/varieties of the same species, pangenome (supragenome) analysis can assist in detecting new genes of the wild relatives. These genes can be incorporated to re-domesticate those cultivars to withstand more stresses. This is also important as substantial damages inflicted by RKNs are extended to other nematicide-sensitive crops, e.g., Egyptian clover and other forage legumes [35]. Because these forages are used as direct feeding diets for livestock, toxic chemical nematicides may not be used. Newly discovered integration of resistant cultivars with BCAs can offer a novel eco-friendly strategy to control RKNs in kiwifruit orchards [36]. Moreover, recently published greenhouse tests demonstrated that grafting may alleviate the problems of these pathotypes. New rotation sequences of grafted tomato-melon-pepper-watermelon on resistant rootstocks could revert the virulent *Meloidogyne incognita* populations on the *Mi1.2* gene and consequently minimize yield losses compared to ungrafted plants [37]. Using resistant rootstocks, both intra- and interspecies grafting can provide safe and effective alternatives to choose and apply desirable PPN resistance trait in cultivated vegetables, whether in open-field or protected conditions. As many cultivars of solanaceous and cucurbitaceous crops are highly susceptible to RKNs, many such cultivar rootstocks need to be commercially available and easily accessible. For PPN control and boosting plant production, factors such as proper scion genotypes, local settings, target/present PPN species/races, and PPN population pressure in the desired cropping sites should be considered. Phani et al. [38] speculated that nematode populations and their dynamics as well as the related soil microbiome community should be monitored, as they may shift due to frequent usage of grafted vegetables under intensive agriculture. Therefore, rotation of rootstocks among various plant species/cultivars is a preferred precautionary measure to avoid the emergence of aggressive/virulent pathotypes in response to selective pressures. Also, epigenetic changes that might affect grafted plants should be continuously explored via transcriptome analyses and functional studies. On the other hand, introducing resilient crops to different cropping systems, e.g., strip cropping or intercropping, may sustain nematode-resistant genes. These may include poor/non-host crops that are suitable to reduce PPN population densities in the soil. Likewise, good weed control is critical, as many weeds are very good hosts [2,8,26].

The manipulation of molecular genetics as a novel strategy for PPN management is quite possible. Incorporating various discoveries and new techniques to illustrate and employ effective mechanisms of natural plant resistance can represent promising tools to advance such strategies [39]. In this context, genetic engineering can also be exploited to widen the gene pools of available plant cultivars and genotypes within the sophisticated plant mechanisms that are utilized to perceive and combat PPN attacks. For instance, the aforementioned breeding programs may be backed with a metabolomics approach. If so, breeders for PPN resistance can evaluate the relevant chemical compounds and secondary metabolites using current chromatographic methods. These methods include liquid chromatography, gas chromatography, nuclear magnetic resonance spectroscopy, capillary electrophoresis, mass spectrometry, and thin layer chromatography [40]. Both stimulated (phytoalexins) and constitutive (phytoanticipins) plant defensive metabolites that possess nematicidal impacts can be defined and measured in breeding programs via such tools. The existence and levels of PPN suppression-induced metabolic compounds such as phenolics, thiophenes, isothiocyanates, alkaloids, glucosides, and tannins can help plant breeders to optimize their programs for improved nematode-resistant genotypes [40]. Moreover, many phytochemicals are used to form robust bases to engineer durable bionematicides. Faria et al. [41] reported that analysis of data on the accumulation of volatile organic compounds (VOCs) on plants from the use of nematicidal essential oils revealed their capability to control the pinewood nematode, *Bursaphelenchus xylophilus*, under the IPM framework and consequently to boost forest health.

Although screening techniques for host suitability designations of various PPN species have been well-established [42,43], further improvements in their methodologies can optimize PPN management strategies. Sasser et al. [44] created a scale to assign the degree of resistance to RKNs that is uniformly applicable to all crops and negates the formerly required standard susceptible cultivars for comparison. Their scale is based on using both the index of RKN galls/egg masses (GI) via a 0–5 rating [43] to express the magnitude of plant damage in addition to the RKN reproduction factor to represent host efficiency. The scale offers a quick and easy process for designating resistant and tolerant cultivars (e.g., GI ≤ 2). Hence, some researchers are still using it to easily compare their results with other studies. However, a substantial deficit of this scale is that the relationship of the GI to yield parameters/productions of many crops has not yet been investigated. Because boosting crop yield is the core of PPN management, the scale should be improved to increase nematode management strategies. Recently, improved host suitability designations of various sugar beet genotypes was recorded by a modified host–parasite index scale, which included quantitative and qualitative yield performance [45]. Consequently, this modified scale upgraded/assigned the sugar beet variety Lammia KWS to the tolerant instead of susceptible category manifested by Sasser et al.’s [44] scale. On the other hand, additional speculations suggest other possible fine-tuning to the widely used scale by Taylor and Sasser [43]. For instance, using RKN egg mass index (EI) is better than gall index (GI) because the EI can define nematode fecundity. Nevertheless, because El cannot adequately scale reproduction, using egg numbers may be superior to EI, GI, or other developmental stages for assessing the sedentary nematode reproduction factor [46]. Refining a classical scale for host suitability designation may be necessary in specific cases for more accurate strategies in novel PPN management programs.

## 4. Bridging the Gap between Current and Novel Strategies for PPN Management

The aforementioned techniques can generally shape the basis of emerging and new strategies for PPN management. Furthermore, bridging the gap between present and new strategies for nematode control might be better addressed via the three following levels.

### 4.1. Upgrading PPN Control via Boosting and Adjusting Agricultural Production Scales

Emerging/novel tactics and strategies can use a variety of improved materials and techniques that encompass novel chemical nematicides [47,48,49,50], more PPN-resistant genotypes [32], additional certified seed/planting material [51], more timely and optimized soil solarization for PPN disinfestation [52], and novel bionematicides [53] along with one or more production practices related directly/indirectly to PPN control. These production practices may include ploughing, crop sequencing, fallowing, cover crops, flooding, and/or soil amendments. Developing these novel materials/techniques is necessary to address major problems [51,54,55]. To satisfy the nematicide market demand, these chemicals/materials should effectively slow or block the growth of PPN resistance and avoid health and ecological hazards while increasing crop yields. Moreover, many synthetic chemicals can effectively work as nematicides [56] though their contact with PPN within plant roots or in the soil rhizosphere [57]. Hence, an ongoing priority area is researching the synthesis/rational design of new chemical nematicides that offer safe, economic, and effective alternatives to unhealthy chemicals.

#### 4.1.1. Exploring Further Nematicidal Compounds

Chen et al. [48] reviewed studies on numerous nematicidal compounds to determine their structure–activity relation and mode of action, and they suggested some nematicidal active fragments. For instance, as heterocyclic compounds play a key role in releasing novel nematicides, the relevant novel nematicides fluazaindolizine, tioxazafen, fluopyram, fluensulfone, cyclobutrifluram, and imicyafos have appeared in the last two decades, while others are in the pipeline. The novel active ingredient fluazaindolizine was registered with the Environmental Protection Agency (EPA) on 12 September 2023 (https://www.epa.gov/pesticides/epa-registers-new-active-ingredient-fluazaindolizine, accessed on 3 June 2024). It is useful for PPN control on many horticultural crops, e.g., vegetables (tomatoes, carrots, taro, squash, potatoes, and eggplant) and fruit trees (citrus, grapes, almonds, and peaches).

Meanwhile, within the recently released group of seed-treatment nematicides such as pyridinyl ethylbenzamides (fluopyram), avermectins (abamectin), and phenetylarylamides (cyclobutrifluram), several authors presented Tioxazafen (NemaStrike™, Bayer, Leverkusen, Germany) as a good example to manifest the group’s merits [47] and called for expansion in their applications. The treated seeds serve to transport the active nematicide ingredient in the rhizosphere to the exact site where it is needed to control PPN attacks on the plants’ roots. An additional merit of seed treatment relative to in-furrow treatment is the cost savings, as the amount of active ingredient applied per unit area is minimized. The first seed treatment offering such merits was Avicta^TM^ (abamectin) in cotton-planted soil in the US in 2006, but it could be expanded to other important crops like maize and soybeans for broad-spectrum control of nematodes [58]. With such a new technique, an additional market segment for field crops was introduced, and most growers refrained from using in-furrow nematicides. Thereafter, seeds could be treated with nematicide as a standalone product or even as a dual-purpose product, e.g., in combination with an insecticide [59].

Novel nematicides with superior traits can contribute to bridging the aforementioned gap. Tioxazafen is a novel class of nematicides as it possesses a disubstituted oxadiazole skeleton that can remain in the plants’ roots for about 75 days during the course of crop growth. Meanwhile, it can selectively inhibit nematodes’ production of cellular energy [47]. Fluopyram demonstrated a high nematicidal efficacy against several serious PPN genera, such as root-lesion nematodes, RKNs, cyst nematodes, and potato rot nematodes. Furthermore, in a 2-year field study to control *Meloidogyne incognita* infecting watermelon, Nnamdi and Hajihassani [49] tried to further optimize the efficacy of three novel nematicides (fluensulfone, fluazaindolizine, and fluopyram), known as 3-F nematicides. They applied them at different times (days before transplanting, at transplanting, and days after transplanting), nematicidal rates, and combinations (including with an old one, oxamyl). Except for the one-time use of oxamyl in both years, the nematicide treatments significantly decreased *M*. *incognita* galls on watermelon roots relative to the untreated control. Similarly, except for a single use of oxamyl in the year 2020, all treatments manifested a lower *M. incognita* population level in soil than the untreated control. Overall, they [49] concluded that drip application of the novel 3-F nematicides demonstrated a profitable option for managing *M*. *incognita* on watermelon. General trends in developing these chemicals are their novel nematicidal function or mode of actions. Therefore, the mechanisms of such new nematicides for controlling nematodes are being studied. While fluensulfone apparently gives rise to gradual metabolic harm that depletes lipid storage, leading to PPN death [48], fluopyram acts as a succinate dehydrogenase inhibitor to suppress mitochondrial complex II of the electron transport chain [60]. Thus, the mode of action of fluensulfone is different from common nematicides like organophosphates and carbamates that inhibit acetyl cholinesterase. Regrettably, a side effect of fluensulfone’s efficacy is that it can cause risky levels of toxicity in non-target organisms. Fluensulfone has further merits, as it could be applied via different methods, e.g., drip irrigation and foliar spraying. Yet, Chen et al. [48] stressed that it can affect PPNs differently depending on the soil type and application method. Another recently explored mode of action is to interfere with ribosomes via tioxazafen usage [61]. On the other hand, a thrilling paper [55] published in *Nature* opened new avenues for boosting novel PPN control. Its authors sifted through their chemical library to find nematicidal compounds to control *Caenorhabditis elegans*. They came up with three novel imidazothiazole-containing molecules that showed nematode-killing activity. They named them selectivin-A, selectivin-B, and selectivin-C. These selectivins could effectively kill not only RKN *M*. *incognita* but also four free-living nematode species as well as a nematode parasite of cattle. More importantly, selectivins showed almost no toxicity concerning non-nematode species such as human cells, fungi, fish, and insects. They concluded that selectivins are more nematode-selective than commonly marketed nematicidal products. These novel compounds are first-in-class bioactivated nematode controls that offer reliability and nematode selectivity; they have a distinguished mode of action relative to the marketed nematicides examined [55]. The authors determined that the mechanisms by which selectivins killed nematodes depends on microsomal cytochrome P450 (CYP) enzymes that are frequently engaged in converting dormant pro-drugs into active compounds [62]. In contrast to other tested organisms, this type of bioactivation was highly specific for nematodes because selectivin-A-derived metabolites are either not found or are severely diminished in such organisms. Specifically, they [55] found that *Meloidogyne incognita* possesses the CYP enzyme CYP4731A3 that bioactivates selectivin-A, causing damage to nematodes.

Other examples are related to novel strategies for PPN management on key crops via bionematicides, i.e., BCAs and/or their bioactive compounds. Bionematicides are mostly void of negatives that are frequently associated with chemical nematicides. They generally have adequate management of resistance-breaking pathotypes, are safe for beneficial organisms, and are free of pollutants or harmful residues [63]. Therefore, continuous efforts to produce reliable and safe nematicidal materials to supplant synthetic chemicals have resulted in a few bionematicides, with market demand for their applications currently growing [53,59,64]. Wilson and Jackson [59] reviewed the active ingredients of four key bionematicide products manufactured by international companies: Nortica^®^ (*Bacillus firmus*), DiTera^®^ DF and DiTera wettable powder (WP) of *Myrothecium verrucaria* fermentation broth, Econem^TM^ (*Pasteuria usgae*), and MeloCon^®^ or BioAct WP (*Purpureocillium lilacinus*). Certain *B*. *firmus* strains can colonize *Meloidogyne* spp. egg sacs and destroy the eggs, resulting in declining numbers of both nematode galls and infectious juveniles [65]. Cell free filtrates of these *B*. *firmus* strains contain toxic metabolites that can induce paralysis and death of larval and adult nematodes of the species *M*. *incognita*, *Radopholus similis*, and *Ditylenchus dipsaci* [66]. As for the fungus *Myrothecium verrucaria* fermentation broth, its commercial products include fungus bioreactor suspension resulting from its in vitro production. Its mode of action is assumed to depend on combinations of ingredients rather than a single toxin. Wilson and Jackson [59] summarized the mechanisms of the active ingredient in the product: it kills PPNs on contact, inhibits eggs from hatching, affects the rate of movement/host finding, and shifts the plant’s rhizosphere microbiota to boost microbial antagonism. As for *Pasteuria*, seven nematode-attacking species of this genus are recognized, but it is plausible that there are many taxa that exhibit sizeable variety at an intra-species level [67]. There are at least four species that can parasitize PPNs in control programs: *Pasteuria penetrans* on *Meloidogyne* spp., *P*. *thornei* on *Pratylenchus* spp., *P*. *nishizawae* on both *Heterodera* spp. and *Globodera* spp., and *P*. *usage* on *Belonolaimus* spp. [58]. Suggested *Pasteuria*-based products currently used to control PPNs in modern ecosystems are presented (Table 1). Because there are no commercial products containing *Pasteuria thornei* endospores, Stirling [67] recently reported the best option to obtain supplies of these endospores via an adaptation of the dried root technique that is broadly used for *Pasteuria penetrans*. *Pratylenchus* species can be in vitro cultured on sterilized carrot tissue. The method includes adding the target nematodes to an endospore suspension taken from spore-filled cadavers and inoculating them with *Pasteuria thornei* spores. Thereafter, the nematodes are inoculated onto a host plant, whose roots are harvested 6–8 weeks later, air dried, and then ground into a powder. However, if the nematodes are still alive in the roots, air-dried soil should be used instead of roots as an inoculum source for emerging and novel integrated nematode management (INM) schemes. Because *P*. *lilacinus* is commercialized as a water suspension, the fungus is applied via irrigation or with a ground application device. The water-dispersible granule (WG) formulation comprises 1 × 10^10^ and the WP formulation contains 1 × 10^11^ fungal spores g^−1^ product. While application methods and times, rates, targeted PPN species, and host plants of such emerging biocontrol methods and BCAs are often found in their product labels and referenced [59,67,68,69,70,71], others are still in the production pipeline or will be available soon. Tables listing fungal and bacterial BCAs utilized with other components in INM, their commercial products, formulation types, and modes of action on PPN-infected economic crops are also presented elsewhere [68].

Commercial bionematicides may also contain plant components. For instance, Nemguard is being marketed as a bionematicide (NEMguard^®^, Ecospray, Alzano Scrivia, Italy). It is registered for soil application to control PPNs based on garlic extract. It has biologically active ingredients such as allicin and polysulfides found in the garlic concentrate. Besides its nematicidal activity, this product possesses both insecticidal and fungicidal properties [47,78]. Although NEMguard WF has been registered for use on a wide range of crops such as carrots, potatoes, and tomatoes, it is still being tested for expansion on other fruit trees such as grape [53]. Very recently, the authors concluded that this product could be a reliable and safe nematicide for dagger nematode (*Xiphinema index*) management in both organic and integrated vineyards. Their conclusion implies that this and other bionematicide products are likely to become widely available soon. Diallyl polysulfides, i.e., the active ingredients of Nemguard^®^, seem to have multi-site activity as hypothesized by Chatterji et al. [79]. They reported DNA damage and/or cell apoptosis of the tested PPNs after the generation of reactive oxygen species by polysulfide materials. Also, disturbance of metal homeostasis was suggested because of the metal-binding capacity of diallyl polysulfides [80].

#### 4.1.2. Harnessing Biotechnology to Comply with Ecological and Biological Factors

The forward-looking concept of upgrading PPN management strategies lends further urgency to the need to optimize other relevant tactics, such as exploiting more botanicals used as bionematicides as well as non- and poor-host crops and nematode-suppressive soils [8,52]. Over and above, several plant industries may be economically served by PPN control tactics. For instance, large-scale implementation of the waste-to-resource concept in the olive sector could exploit olive mill wastes for controlling serious pests such as PPNs [81]. Furthermore, it is imperative to characterize and grasp closely related factors that usually interact and affect such PPN management strategies. As most of these factors have already been addressed (see [8,39,50,52]), a brief update is necessary. A better grasp of the nature of PPN–host interactions that regulate plant susceptibility/resistance can boost the discovery and use of PPN-resistant genes. Also, expansion of metagenomic tools and artificial intelligence for emerging PPN control plans will supply stakeholders with swifter, more accurate PPN identification and decision tools by properly revealing the food web in the plant rhizosphere and the exact mechanisms underlying PPN-suppressive soils [13,25,37,39,52].

Recently, a chapter published by Helder and Heuer [82] entitled “Let’s be inclusive—the time of looking at individual plant parasitic nematodes is over, and new technologies allow for it” discussed various modern approaches that should be integrated and exploited. Admittedly, disease complexes engaging multiple nematode genera/species/races are very common globally. Hence, current PCR-based technologies and phylogenetics should be exploited not only by accurately demonstrating patterns of development and diversification among plant pathogens but also by assessing their associated beneficial/harmful microbiota in the plant’s rhizosphere [23,24,83,84,85,86]. For instance, there was no previous single-tube assay that could detect *Meloidogyne fallax*, *M*. *chitwoodi*, and/or *M*. *minor* in a sample, but advanced molecular techniques can reliably do this from crude PPN extracts [86]. Interestingly, PCR-based technology also indicated a highly significant spatial distribution link between *Fusarium solani* (a plant pathogen) and polyspecific EPN (biocontrol agents of insect and PPN pests) communities in a 6-month citrus grove survey [87]. Likewise, ecological factors should be included when exploring novel strategies for PPN control and IPM, especially on key crops. Unlike conventional practices in citrus orchards in Florida (USA), an advanced production system (APS) fertilized trees daily to avoid a negatively affected yield from citrus greening disease. Consequently, APS changes the soil properties in ways that affected soil biota as determined by real-time qPCR in different experiments. Indigenous EPN numbers were decreased via APS, whereas nematophagous fungal reactions to EPNs relied on EPN species [88]. Therefore, such new technologies should not neglect considering basic factors that affect the success of BCAs against PPNs [89]. They should appraise both the influence of the control measure(s) on the PPN population within mostly multiplex crop production regimes and the cost–benefit ratios for farmer acceptance [90]. Truly, important variables can offer insights into how soil properties can be utilized to optimize PPN control measures [29]. Key factors such as soil moisture and texture [91], pH [88], salinity [92], mulching [93], and disease complexes involving multiple PPN species [94] have been found to modify nematode population levels directly or indirectly by impacting their hosts and/or predators [23]. Thus, growers and stakeholders should also target nematicidal application times, as feasible, to harness favorable natural conditions, e.g., precipitation to boost management outcomes. As such factors can often positively/negatively interfere by modifying nematode–nematode interactions, these interactions should be studied on a case-by-case basis [39].

#### 4.1.3. Adjusted Agricultural Practices and Techniques

Despite the close relationship between the above-mentioned biological/ecological factors and the agricultural exercises that comply with them, there are other practices that can more directly target effective and safe PPN control. Hence, progress in basic approaches to develop PPN management strategies should be expanded by incorporating new technologies. For instance, given the above-mentioned benign mechanisms of emerging nematicides, their applications should be expanded to act more reliably. In this vein, unfavorable levels of toxicity from classical chemical nematicides that are still in use should be lowered via adjusted agricultural practices. Various techniques to minimize their doses via co-application with other control measures for their synergistic/additive results have been recorded [8,68]. Numerous such examples of simultaneous usage as well as sequential and dual-purpose application of nematicidal inputs for enhancing their safe application and improving INM have been reported [8,46,54] and should be expanded. Besides, new technologies of nanoparticles (NPs) should further be integrated into PPN management strategies. They can be employed not only as delivery devices for genetic material and probes for emerging biocontrol strategies but also as biosensors for nematode diagnostics [95]. Their relevant nematicides possess functional merits due to their minute size, wide surface area, and raised reactivity of their related NPs. Numerous forms of nano-nematicides could manifest both big biocompatibility and high efficiency in PPN control. They may reduce nematicidal costs while minimizing both environmental pollution and applied doses of nematicides [96]. A nano-nematicide that can easily reach the deep root zones of plants to kill most PPNs has been reported. Nano-nematicides are leading a substantial shift in PPN control, but recent recommendations have emphasized the need to secure both their clean manufacturing and usage with cost-effective techniques [97].

Other novel technologies using meta-DNA barcoding and next-generation sequencing are being incorporated to reliably assess various nematode communities [25,82,83,84,85,86,87]. Wesemael and Visser [98] speculated that these technologies will enable us to adjust our agricultural exercises to reduce crop losses caused by nematode pests. Among such novel strategies, for example, these authors expected that plant breeding schemes will generate crops with resistance to key PPNs, which would give more options for rotation and supplementary benefits of cover crops and cover crop mixtures used in the control strategies. Introducing novel functional genetic devices will help improve the understanding of plant–nematode interactions to develop more crops with resistance to key PPNs via genome editing [99]. However, at high PPN infestation densities, especially for RKN-susceptible crops such as many cucurbits, growers will still have to utilize chemicals or shift to soilless cultivation [100]. Other practices such as tillage, irrigation, weed control, fertilization, and soil amendments must be manipulated at the appropriate time to prepare the soil environment to enhance and/or maintain BCAs for conservation biocontrol [101]. Meanwhile, exploring the spatial distribution of nematode pests in the field will improve sound management decisions [102] and lead to exact practicability of site-specific nematicide application to lower the required dosage [28]. In other words, it is still quite possible to include chemical nematicides as components within the INM given the aforementioned tactics [49,52,68] and precautions [103]. Such precautions of fumigants in novel PPN control strategies impose restrictions in terms of enlarged buffer areas, localized decreases of VOCs being freed into the atmosphere, and proper personal equipment to protect field workers [103]. Emerging alternative techniques to these fumigants are being explored. These may comprise new biological soil disinfestation approaches backed by soil solarization to suppress multiple pests/pathogens [104,105] and developing PPN-suppressive soils [8,29,52].

### 4.2. Bridging the Global North–South Divide for Accessing Extensive PPN Management Plans

An additional challenge is the discrepancy in technological advances for nematode control between the well-developed and developing countries [90,101]. While management measures for PPNs in tropical and subtropical countries are lagging behind those in the US and most European countries, rapid progress in nematology-related techniques, such as applications of molecular diagnostics, remote/proximal sensing to detect/identify PPN infestation, and biochemical/molecular taxonomy to quantify various PPN species and their related microorganisms, may widen the gap between the two areas. For instance, difficulties detecting emerging and new PPN threats, especially with expected changed in related climate factors in sub-Saharan Africa, may impede the timely employment of PPN control strategies and aggravate crop yield losses [1,90]. Basically, laboratories/centers with frontiers in PPN diagnostics, systematics, and management programs like those in developed countries should be expanded to less developed ones. Emerging isothermal amplification techniques and remote sensing approaches are being introduced for PPN diagnoses on the spot (in the field) that are swift, accurate, and affordable. Yet, using integrative diagnoses that merge remote sensing to molecular techniques is more adequate in the field [106]. Tackling PPN problems via such novel technologies should continue to be expanded within worldwide IPM programs, especially in heavily PPN-infested areas. Therefore, the following major schemes should be earnestly attempted.

#### 4.2.1. Integral Perspectives and Partnerships for New Technologies

After banning effective but unhealthy chemical nematicides such as aldicarb [107] and carbofuran [108], strategies to cut back on further similar chemicals have spurred interest in developing reliable, inexpensive, and ecologically benign alternatives to these chemicals. Therefore, all various PPN-related disciplines should cooperate to develop traditional or novel PPN management plans. They should support the main pillars of INM, namely, cultivar choice/setup, crop sequence, control decisions, soil management, and monitoring/estimation [51]. The authors of [51] detailed various PPN control tools and devices that construct each of these pillars to work in perfect and integral harmony. Obviously, the applicability of these tools varies according to nematode–plant interactions, production zones/regions, and availability of materials and techniques around the world. Nonetheless, maximum/perfected standardization in nematode-related procedures should be attempted whenever possible. This is especially important for common processes for both researchers and stakeholders. For example, a standardized nematode sampling tool can grant a sound comparison between different trials and expand the analysis of individual trials for perfection of conclusions [10]. Moreover, differences in the size of the sampling unit may lead to variable indices of nematode dispersion with consequent misinterpretation of the data [109]. Because it is practically impossible for nematologists and related researchers to act united, they must utilize mutual/common procedures that ease future evaluations and allow them to be built/expanded on. Such strategies will also enable them to face many challenges related to the current and emerging nematicidal materials and techniques, such as their possible inherent nature (e.g., high volatility and lipophilicity of some essential oils as nematicides), costs of production and application, and manufacturing constraints. To overcome these constraints, intimate cooperation among researchers of various disciplines/countries is expected. They can integrate PPN control measures with emerging innovations such as the bioinformatics [110], soil microbiota [111], volatile organic compounds as cues to strengthen biocontrol strategies of pests [112], nanoencapsulation of nematicides [113], and RNA-Seq in biopesticide development [39,110,114]. PPN management strategies motivated by bioinformatics can map the reactions of the active ingredients (AIs) in biopesticides with their target pests to tackle multiple diseases [114]. Abd-Elgawad [28] recently reviewed CRISPR-PN2, which is a software program (CRISPR-Cas9) that uses a genome-aware platform to assess genetic assays on the target nematodes. It offers resilient applications and commands over the automated drafts of certain pilot RNA sequences for CRISPR tests on parasitic nematodes. The program is also used to back gene editing at the fixed scale. Surely, this gene editing can assist in enhancing the desirable traits of plants that do not have enough PPN-resistant genes. It could overexpress the genes commanding the biocontrol process exerted by *Paecilomyces javanicus* to boost plant protection against infection with RKNs [115]. Merits and demerits in nanopesticides with controlled-release attributes were recently discussed to indicate a few constraints and how to avoid them by optimizing the response of nanocarriers to ecological and biological stimuli such as temperature, pH change, redox conditions, light, and the enzymes present [116]. Fortunately, their delivery is still done in very small amounts of active substances to avoid environmental pollution [97,117]. Additionally, merging such advanced technologies into nematode distribution patterns to upgrade biological control of PPNs and other pests has recently been proposed in detail [28,113]. Moreover, olfactory signals emitted from VOCs that are generated by certain plant species can be involved in PPN control tactics. Silva et al. [118] reported that water subjected to VOCs from *Piper nigrum* (L.), *Cymbopogon nardus* (L.), and *Bertholletia excelsa* (Bonpl.) reduced the motility of *Meloidogyne incognita* second stage juveniles (J_2_) by 42%, but VOCs of *Brassica oleracea* (L.) induced almost full immobility. Likewise, VOCs from *B. oleracea* shoots killed the J_2_ and decreased the number of *M*. *incognita* galls and eggs on tomato roots. Also, dimethyl disulfide and 3-pentanol obtained from *B*. *oleracea* volatilome [119] reduced egg hatching by 96.8% at 176 mg/L and by 88.4% at 918 mg/L. Because J_2_ and eggs are basic stages in the life cycle of RKNs, such VOCs should be developed into novel nematicidal products. In parallel with such cutting-edge technologies, well-designed programs should address various aspects to raise growers’ awareness and extend crop protection. Cooperative programs on a country level and between countries may be set up that comprise relevant information for stakeholders to incorporate new knowledge/technologies into modern farm management, including PPN control. For these programs, training farmers/extension officers about the above-mentioned pillars of INM as well as their proper usage and effects on beneficial/harmful microorganisms, crops, and soil health should be an integral and imperative component of the holistic strategies for pest management.

#### 4.2.2. Expansion of Effective Decision Support Tools and Decision Support Systems

Upgrading PPN control strategies is knowledge intensive and should combine all modern and traditional components related to effective and sustainable PPN management worldwide. These comprise nematode population level and distribution, crop sensitivity, host status, and damage thresholds with known efficacy of individual management measures [120]. Therefore, modern structures of agricultural plans for pest management usually have both decision support tools (DSTs) and decision support systems (DSSs). Such tools and systems are established or being evolved mostly in developed countries via scientists working in both governmental and private sectors. These tools can produce information on certain abiotic factors that impact PPN behavior in the field. Consequently, they can be used to optimize INM. Molendijk and Sikora [120] reported tools such as soil sensors (e.g., Nematool and LoRa Soil sensor), remote sensing, and predictive models. Also, DSS can assist farmers and stakeholders in making real-time decisions for appropriate farm management, which may involve PPN problems. Related programs can merge information on numerous different features necessary for improved farm management. Being holistic, these DSSs are not necessarily centered around INM. For instance, some of these DSSs have been developed by the European Union in order to improve similar plans for other PPNs in adequate cropping systems worldwide [120]. Among them, Akkerweb/FarmMaps offers the information needed for practical smart farming, such as immediate data about weather services, soil maps, satellite crop images, and crop polygons [121]. Its related software programs also provide an array of models, a DSS digital store, visualization tools, and a task map generator. Other DSSs for managing PPNs are included in NemaDecide [122]. Various relevant models/equations for population dynamics of *Globodera* spp., *Meloidogyne chitwoodi*, and/or *Pratylenchus penetrans* on potatoes, soil sampling, yield loss, partial resistance, and control measures are provided by NemaDecide. NemaDecide integrates data models/sources to serve strategic decisions at the farm/field level. Thus, NemaDecide supplies useful estimations of needed variables such as risks for possible potato crop loss, the possibility of PPN population growth, level of probability for detecting PPN foci by soil sampling, and assessing costs/benefits of control measures. Thus, it offers advice for growers and stakeholders to improve financial returns and compare different cropping scenarios. Also, the combinatorial impact of several biotechnological tools for accurate PPN detection as a prerequisite for INM plans should be exploited and expanded. For instance, magnetic biosensors combined with a DNA extraction protocol and loop-mediated isothermal amplification technique can form a novel solution for in-field diagnostics of the serious nematode *Globodera pallida*. This method possesses the capacity to detect as low as only one *G*. *pallida* juvenile, even when found with other closely related PPN species [123].

### 4.3. Harmonization of Nematicidal Operations and Their Novel Targets

The aforementioned coordination should include not only nematicide operations but also pesticides in general, especially for integrated pest control purposes. An emerging example is a new nematicide that can effectively target nematode-chitin deacetylase. Due to chitin-related proteins found in soilborne fungi and PPNs, nematode-chitin deacetylase inhibitor (CDI), namely N2-(dimethylsulfamoyl)-N-{2-[(2-methyl-2-propanyl)sulfanyl]ethyl}-N2-phenylglycinmide, can work against both groups of plant pathogens [124]. Fortunately, CDI can target many chitin-rich animal tissues of additional plant pathogens and pests, such as the exoskeletons of arthropods [125]. Its nematicidal application has several merits, as it has low toxicity to humans and wildlife, with the exception of insects. Its compounds also possess a fading environmental effect, as they degrade fast and do not amass in water or the soil [124]. Such strategies can be used to manage pesticide use and run-off as well as achieve safe agricultural production. Obviously, the stability and effectiveness of cooperation mechanisms among all partners will vary under various scenarios. Therefore, Gong et al. [126] recently established definite computer models to identify certain equilibrium strategies. The models mainly comprise different interactions among the growers, government, and pesticide operators as crucial elements that influence the effectiveness of pesticide regulations. Among these elements, the robustness and stability of actions to encourage growers’ oversight, the oversight done by government authorities, the intensity of penalties practiced on non-compliant pesticide operations, the availability of DSTs and DSSs, and the related trust-building measures between all partners (pesticide operators, government, and farmers/stakeholders) are key factors. These models [126] concluded that as these factors hit a definite threshold, cooperation between these partners can result in stabilization of pesticidal strategies and fulfill social optimality. The dire need to accelerate agricultural production without further environmental pollution from using unhealthy nematicides [127,128] bodes well for such model-based strategies [126] via incorporating novel technologies [129] to achieve the improved INM plans envisioned herein. Moreover, manifold deep learning object diagnostic models could be used to provide prompt and informed PPN management strategies while offering a device to exploit broadly shared data and implement their meta-analyses [130].

On the other hand, as exudates of plant roots are key factors in PPNs’ attraction to their hosts, recent molecular tools revealed that the natural nematode surface coat could be regarded as novel targets for nematode management. In this respect, plant growth-promoting bacteria (PGPB), among other biotic/abiotic factors, can significantly affect parasitism of serious nematode groups, e.g., RKNs. Recently, Habteweld et al. [131] reviewed various aspects of tripartite (plant–RKN–PGPB) interactions for incorporation in integrated RKN management strategies.

## 5. Conclusions

With the striking figures regarding PPN losses on many key crops, there is a growing interest in exploiting all aspects for their effective and safe control. Hence, it is concluded that upfront procedures to control PPNs must be improved, especially with the continuing advances in relevant technological interventions. In-depth procedures of the sampling, extraction, identification, and counting principles should be updated to advance PPN control techniques. This should be followed by exploiting numerous current and emerging technologies with an end in view to upgrade nematode management. Advanced synergistic and additive inputs for PPN control, more developed PPN-resistant genotypes, expanded functional plans for nematicidal targets, and bridging the gap between present and new PPN control strategies are envisioned for INM plans. Also, PCR-based approaches are being developed to characterize BCA biogeography and should be harnessed to wisely augment and conserve biocontrol via a fine-scale taxonomic resolution. With the ever decreasing costs of RNA sequencing and data processing, PCR-based approaches will substitute their corresponding classical methods, especially in developing countries. Hence, the availability of more eco-functional transcripts of soil biota along with advances in other related areas such as bioinformatics and nanotechnology should lead to the development of novel PPN management strategies. Furthermore, partnerships among researchers and various entities to test the new relevant technologies as well as regulated pesticide operations should contribute to establishing model-based optimization strategies. Ultimately, developing cost-effective, safe, and reliable nematicides, especially for IPM schemes, can lend further insistence to the call to continuously optimize PPN management strategies for sustainable agriculture. A multifaceted PPN management strategy should merge classical methods with cutting-edge techniques backed by a robust foundation of research, education, and international collaboration.

## Figures and Tables

**Table 1:** *Pasteuria* species used in the management of plant-parasitic nematodes and their available products *.

*Pasteuria* Species (Product Name)	Company (Country)	Target Nematodes	Modes of Action	References
*Pasteuria penetrans* (Econem)	Nematech (Japan)	*Meloidogyne* spp.	Root invasion is diminished with the sterilized nematode host due to the spores attaching to nematode juveniles.	[67]
*Pasteuria* nishizawae (Clariva PN)	Syngenta (Brazil)	*Globodera* spp., *Heterodera* spp.	Bacteria adhere to the cuticle of juveniles to parasitize on them and thus decrease nematode feeding/reproduction.	[72,73]
*Pasteuria thornei*	NA	*Pratylenchus* spp.	Bacteria attach to the nematodes during migratory stages and parasitize them, reducing their feeding/reproduction.	[74,75]
*Pasteuria* sp. Ph3 (Naviva ST)	Syngenta (USA)	*Rotylenchulus reniformis*	Inhibits *R*. *reniformis* in cotton, soybean, vegetables, cucurbits, and floriculture	[76]
*Pasteuria usgae* Bl1 + *Pasteuria* sp. Ph3 (NewPro)	Syngenta (USA)	Species of *Hoplolaimus* and *Belonolaimus*	Inhibits species of *Hoplolaimus* and *Belonolaimus* in turf (Bermudagrass and St. Augustine grass)	[76]

* Modified from [77]. Modes of action are generally similar, but since bacterial species are obligate endoparasites, slight differences may exist due to the targeted biology of the nematode species (e.g., cysts vs. root galls and migratory vs. sedentary species). NA: Not available.

## Data Availability

The original contributions presented in the study are included in the article, further inquiries can be directed to the corresponding author.
